# 

**Published:** 1907-10

**Authors:** 


### CONTENTS.

ORIGINAL COMMUNICATIONS—

The Treatment of Pueperal Infection. By Geo. H. Noble, M. D. Atlanta, Ga. ........ 38[illegible]

Alcoholism and Tuberculosis. By Louis C. Rouglin, M. D., Atlanta, Ga. ........ 40[illegible]

Intelligence of Action. By C. A. F. Lindorme, Ph. D., M. D.. Atlanta, Ga. ........ 405

An Operation for Hemorrhoids Practically Painless and Bloodless. By R. R. Kime, M. D., Atlanta, Ga. 41[illegible]

Leptandrin. By James Burke, M. D., Manitowoc. Wis. ........ 42[illegible]

Some Observations on Surgical Dressings and the Care of Wounds. By J. H. Johns, M. D. ........ 42[illegible]

CORRESPONDENCE ........ 425

NEWS AND NOTES ........ 427

EDITORIAL NOTES AND COMMENTS—

Report of the Henry Phipps Itstitute ........ 431
The Medical Ideal ........ 432

BOOK REVIEWS ........ 434

SELECTIONS AND ABSTRACTS—

The Deposition of the Appendiceal Stump. By Benjamin Merrill Ricketts, Ph. B. M., LL. D. ........ 437
Puerperal Toxemia ........ 438

MEDICAL ITEMS

The Greatest of Anesthetics ........ 441
Gastro-Intestinal Ailments of Young Children. By B. Brown, M. D., Waukegan. Ill. ........ 442
The Use of Adrenalin During Ether Anesthesia. By Charles S. Venable, M. D., Charlottsville, Va. ........ 444
The Cure of a Case of Osteomalacia ........ 445

